# Pulmonary endarterectomy in patients with occlusive isolated pulmonary vasculitis

**DOI:** 10.1186/s40001-023-01239-8

**Published:** 2023-08-07

**Authors:** Jiexu Ma, Wu Song, Hang Xu, ZhaoJi Zhong, Yige Huyan, Sheng Liu

**Affiliations:** 1 Department of Cardiac Surgery, Key Laboratory of Pulmonary Vascular Medicine, Fuwai Hospital, National Center for Cardiovascular Diseases, Chinese Academy of Medical Sciences and Peking Union Medical College, No. 167, Beilishi Road, Beijing, 100037 China; 2 Department of Cardiovascular Surgery, Union Hospital, Tongji Medical College, Huazhong University of Science and Technology, No.1277, Jiefang Avenue, Wuhan, 430022 China

**Keywords:** Pulmonary vasculitis, Pulmonary endarterectomy, Pulmonary hypertension, Chronic thromboembolic pulmonary hypertension, Immunosuppressive therapy

## Abstract

**Background:**

Isolated pulmonary vasculitis (IPV) is a rare, insidious, and localized inflammatory disease affecting the pulmonary arteries, often leading to severe luminal obstruction. The prognosis for patients with occlusive IPV is poor, and there is currently a lack of effective treatments. The objective of this study was to evaluate the performance of pulmonary endarterectomy (PEA) as a treatment for occlusive IPV.

**Methods:**

This single-center retrospective analysis included patients who received PEA for occlusive IPV between January 2018 and June 2022. Clinical characteristics and hemodynamic parameters were evaluated at baseline and follow-up.

**Results:**

Among 114 consecutive patients who underwent PEA, occlusive IPV was identified in 7 patients. Two patients underwent bilateral PEA for the involvement of both pulmonary arteries. Patch angioplasty was performed to treat four severe constrictions. One patient died from residual pulmonary hypertension after limited PEA of a transmural vascular lesion. In addition, no obvious surgical complications were observed. Three months after PEA, a substantial relief in symptoms was achieved. Also, there is a decrease in the mean pulmonary artery pressure (median 33 [20–48] mmHg before versus median 21 [16–26] mmHg after; *P* < 0.018) and pulmonary arterial resistance (median 234 [131–843] dyn.s.cm^−5^ versus median 180 [150–372] dyn.s.cm^−5^; *P* = 0.310). Three patients experienced a relapse of restenosis of the treated arteries within a 6-month follow-up period, despite daily oral prednisolone administration. They were treated with balloon pulmonary angioplasty of both the main pulmonary arteries and branches.

**Conclusions:**

PEA is a valuable choice for treating occlusive IPV, with notable hemodynamic and clinical advantages. To increase long-term vascular patency, complete management should be optimized.

## Background

Isolated pulmonary vasculitis (IPV) is a subtype of localized, single-organ vasculitis that can be distinguished from systemic vasculitis with exclusive involvement of pulmonary arteries. It is a rare inflammatory disorder characterized pathologically by vascular destruction with cellular inflammation and manifested as pulmonary stenosis or occlusion [1]. The incidence and underlying cause of IPV remain unclear because it often mimics chronic thromboembolic pulmonary hypertension (CTEPH). The true diagnosis is based on surgical specimen biopsy and the exclusion of systemic involvement, rather than preoperative assessments based on clinical manifestations, laboratory tests, and imaging [2]. This infers a high likelihood of long-term underdiagnosis. The lack of effective therapies for IPV has been demonstrated by the limited evidence derived from case reports and short case series [3, 4].

Pulmonary endarterectomy (PEA), a surgical procedure that has undergone revisions over the past several decades, is the cornerstone of curative therapy for CTEPH, an obstructive illness that leads to precapillary pulmonary hypertension (PH) [5]. Although both IPV and CTEPH are categorized as the cause of group 4 PH with certain similarities in pathophysiological features, microstructures point to important differences [2]. However, experience with PEA in occlusive IPV is limited to isolated instances in different centers [6]. Whether PEA in IPV might perform as well as PEA in CTEPH remains debatable. In this study, we aimed to determine whether PEA is safe and effective in patients with occlusive IPV.

## Methods

### Ethical statement

The Institutional Review Board of Fuwai Hospital approved this study, and the requirement for informed consent was waived (No. 2018‐991).

### Patient population

At Fuwai Hospital, 114 consecutive patients underwent PEA between January 2018 and June 2022. The surgical specimens from these patients were subjected to histopathological examination. Among them, seven patients were diagnosed with IPV, 95 patients were diagnosed with CTEPH and 12 patients were diagnosed with pulmonary artery sarcoma. The diagnostic criteria for identifying IPV included (1) histopathological findings indicating inflammatory infiltration of the pulmonary arteries, and (2) exclusion of possible association with other organs or vascular involvement [1]. Detailed patient information was obtained from the hospital medical records. They were examined to determine the clinical, pathological, surgical, and mid-term results.

### Preoperative evaluation

Preoperative assessment was carried out by a multidisciplinary team that included rheumatologists, radiologists, cardiologists, and surgeons. The team also developed the patient treatment plans. Computed tomography, ultrasonography, and blood tests were conducted to further examine vascular involvement. To assess pulmonary artery (PA) lesions, respiratory and cardiac functions, and overall health of patients, computed tomography pulmonary angiography (CTPA), right heart catheterization, ventilation–perfusion scan, transthoracic echocardiography, 6-minute walk test, pulmonary function test, and blood gas analysis were performed. Serum indicators, including erythrocyte sedimentation rate (ESR) and C-reactive protein (CRP) level, were used to measure the inflammation degree. Aggressive therapies were discontinued if emergency action was not immediately required in the presence of increased inflammation levels, without evidence of other illnesses or suspected active vasculitis. The indications for PEA were as follows: (I) patients’ symptoms of the respiratory or cardiac systems were attributed to PA abnormalities in the absence of disorders with ambiguous symptoms or diagnostic criteria; (II) New York Heart Association (NYHA) functional classes II–IV; and (III) surgically accessible stenosis or obstruction in the main, lobar, segmental, or subsegmental PA confirmed by CTPA.

### Surgical techniques

Similar to the regular CTEPH operations carried out by the University of California-San Diego Medical Center and as previously described [7–10], all PEAs were completed by the same senior, experienced surgeon (S Liu), who has performed more than 30 PEAs annually in the past 5 years. In brief, following aortic and venous cannulations, cardiopulmonary bypass was initiated, and cooling commenced. Subsequently, the aortopulmonary septum was excised. The ascending aorta and superior vena cava were thoroughly mobilized for right PA lesions. Deep hypothermic circulatory arrest was implemented after the body’s core temperature reached 20 °C. Depending on the variety of lesions, an incision was made longitudinally in the PA direction. The incision might be expanded toward the lower lobe branch, up to 1–2 cm distal to the upper lobe branch’s takeoff. Any new thrombus at the proximal PA was eliminated, followed by careful identification of the endarterectomy plane. The intima was progressively separated until the branches after being annularly peeled from the media, starting at the proximal PA. Finally, if the main PA constricted and became narrow, arteriotomy was repaired using an autologous pericardial patch; otherwise, a running suture was sufficient.

### Post-PEA treatments

Following surgery, anticoagulation with unfractionated heparin was commenced within 12 h, bridging warfarin to achieve a target international normalized ratio between 2.0 and 3.0. Regardless of ESR or CRP values, all patients were treated with intravenous methylprednisolone (80 mg/24 h for 3 days), followed by prednisone (60 mg/day), which was gradually tapered off but maintained. During follow-up, which ended on September 30, 2022, all patients were contacted via phone or in-person visits. For assessment of disease progression and surgical effectiveness, symptoms, NYHA function class, blood tests, echocardiogram, and CTPA image were all evaluated. Three months after surgery, the patients were advised to undergo right heart catheterization. After discussion with the multidisciplinary team and under the direction of a skilled surgeon, balloon pulmonary angioplasty (BPA) was considered if PH and symptoms remained as a result of recurrent or persistent stenosis on PA.

### Statistical analysis

Statistical analyses were performed using SPSS version 22 (SPSS Inc., Chicago, IL, USA). When the data distribution was skewed, continuous variables were summarized as median with interquartile range (IQR: 25th–75th percentiles). Frequencies were used to summarize categorical data (percentages). For comparisons between pre- and post-operative measures, Wilcoxon’s signed-rank test was performed to compare continuous or ordinal variables. All tests were two-tailed, and statistical significance was set at *P* < 0.05.

## Results

### Baseline characteristics

Table 1 provides an overview of the demographic and clinical characteristics of the patients. The median age at the beginning of the symptoms and the diagnosis of IPV were 40 years (IQR: 35–43 years) and 42 years (IQR: 38–44 years), respectively, among the seven patients with recognized IPV. Five patients (83%) were female. Dyspnea and chest tightness were the most prevalent symptoms, and hemoptysis and chest discomfort were also reported. Majority of the patients were NYHA class functional II or III. All patients, except for two (cases 1 and 5), had PH of variable severity. One of the patients (case 2) received tadalafil plus ambrisentan, the medication specifically prescribed for PH. All patients consented to CTPA, which showed decreased pulmonary perfusion caused by considerable stenosis or blockage of the unilateral or bilateral main pulmonary arteries and their branches.

**Table 1:** Baseline characteristics of the patients

Characteristics	Value
Age at initial symptoms, years	40 [35–43]
Age at diagnosis of IPV, years	42 [38–44]
Diagnosis delay, months	14 [10–40]
Body mass index, kg/m^2^	25.4 [18.3–26.7]
Female	5 (71)
Dyspnea	7 (100)
Hemoptysis	4 (57)
Six-minute walking distance, m	472 [407–534]
NYHA functional class	
II	2 (29)
III	5 (71)
NT-pro BNP, pg/mL	92.1 [42.2–266.0]
C-reactive protein, mg/L	2.5 [1.6–8.8]
Erythrocyte sedimentation rate, mm/h	3.0 [2.0–10.0]
Hemodynamics	
Mean right atrial pressure, mm Hg	4 [3–6]
Systolic pulmonary artery pressure, mm Hg	49 [26–78]
Mean pulmonary artery pressure, mm Hg	33 [20–48]
Cardiac index, L/min/m^2^	3.2 [2.9–4.6]
Pulmonary vascular resistance, dyn.s.cm^−5^	234 [131–843]
Medications	
Anticoagulation	7 (100)
PH‐targeted treatment	2 (29)

Data are presented as median [25th–75th percentiles] or number (percentage)

*IPV* isolated pulmonary vasculitis, *NT-pro BNP* N-terminal pro-B-type natriuretic peptide, *NYHA* New York Heart Association, *PH* pulmonary hypertension

### Perioperative details

Table 2 provides a summary of patients’ postoperative information. Intraoperative exploration as revealed by CTPA, showed that the implicated PA wall was concentrically thickened, and the lumen was completely blocked. Due to the substantial narrowing of the main PA, PEA was performed, and in four patients, additional widening of the PA with an autologous pericardial patch was applied. The oval patch was stitched to the edge of the arterial incision using a running suture, most likely from the left or right pulmonary artery, starting until the boundary with the pleural membrane. Except for two (cases 4 and 7), all PEAs were conducted unilaterally, including one that was restricted to the primary right PA (case 2) because of severe transmural arterial fusion. The entire period of circulatory stoppage for a single, unilateral PEA was approximately 20 min. Ten days after surgery, the patient who had undergone limited PEA died from right heart failure caused by lingering PH, while no other serious problems occurred.

**Table 2:** Surgical details and outcomes

Pt. no.	Surgeries	CPB (min)	ACC (min)	DHCA (min)	Postoperative stays (d)	NYHA FC		mPAP (mm Hg)		PVR (dyn.s.cm^−5^)		Follow-up (months)	BPA (times)/lesions (n)
						Pre-PEA	Post-PEA	Pre-PEA	Post-PEA	Pre-PEA	Post-PEA		
1	PEA	129	46	16	8	3	2	19	16	131	174	54	3/17
2	PEA	158	61	3	···	3	···	48	34	1230	489	death	No
3	PEA + patch	253	84	25	14	2	1	25	15	242	372	28	1/1
4	PEA + patch	161	67	18	8	3	2	51	21	843	271	26	5/···
5	PEA + patch	231	122	58	12	2	1	20	18	110	180	17	1/1
6	PEA + patch	214	104	23	7	3	1	33	26	234	150	9	No
7	PEA	230	71	24	15	3	1	33	21	227	145	3	No

*Pt. No.* patient number; *CPB* cardiopulmonary bypass; *ACC* aortic cross-clamp; *DHCA* deep hypothermic circulatory arrest; *NYHA FC* New York Heart Association functional class; *mPAP* mean pulmonary arterial pressure; *PVR* pulmonary vascular resistance; *BPA* baloon pulmonary angioplasty; *PEA* pulmonary endarterectomy

**Fig. 1:**
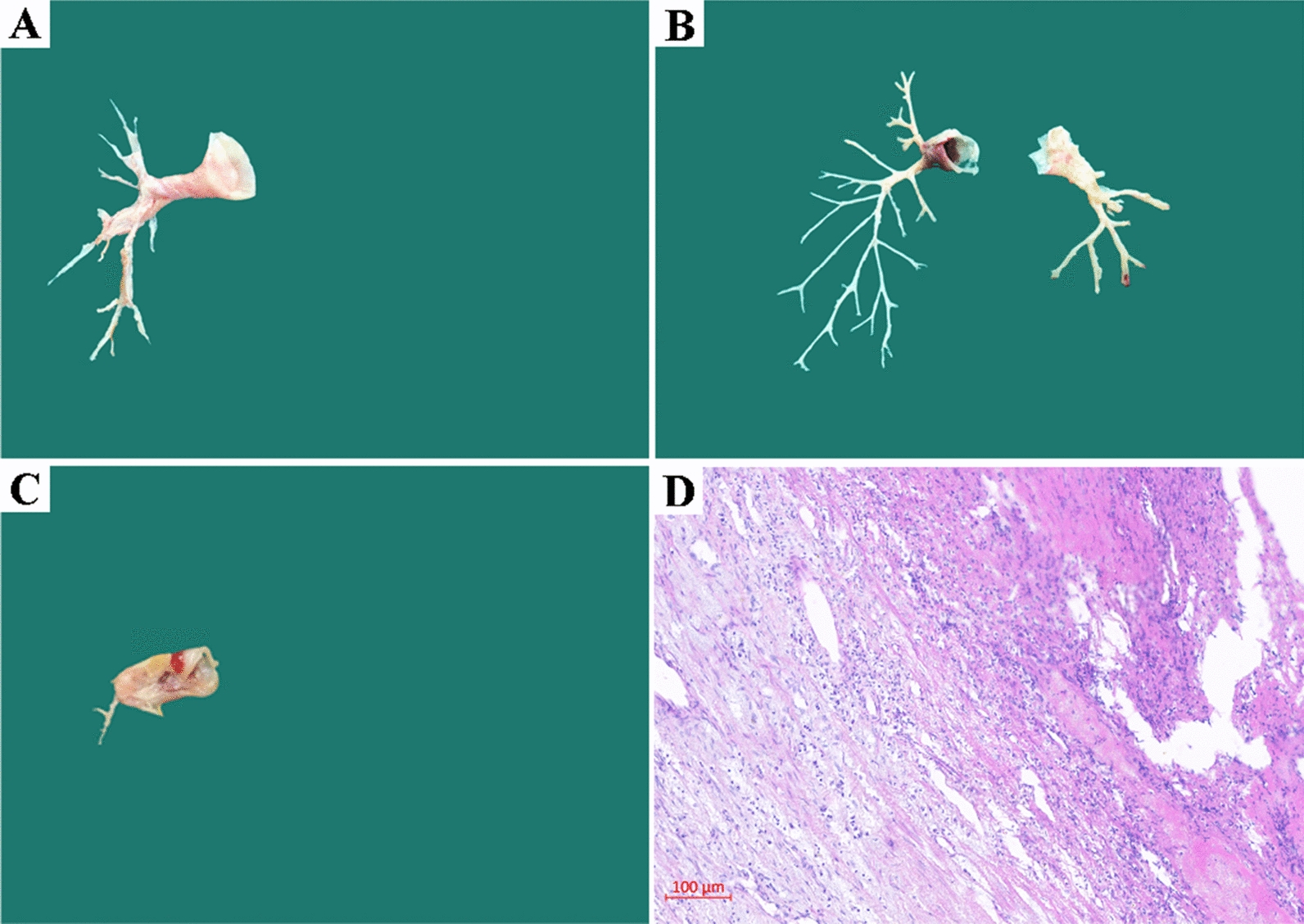
**A**, **B** Representative pulmonary endarterectomy specimens; **C** Specimen of limited pulmonary endarterectomy from case 2. **D** Pathological imaging of endarterium revealed fibrous hyperplasia and widespread lymphocytic infiltration

The PEA specimens are shown in Fig. 1A–C, including the representative endarterium of the main PA and side branches. Diffuse lymphocytic infiltration and fibrous hyperplasia in the artery wall were consistent findings on pathological examination performed following PEA, indicating the presence of persistent inflammation and being characterized as vasculitis (Fig. 1D).

**Fig. 2:**
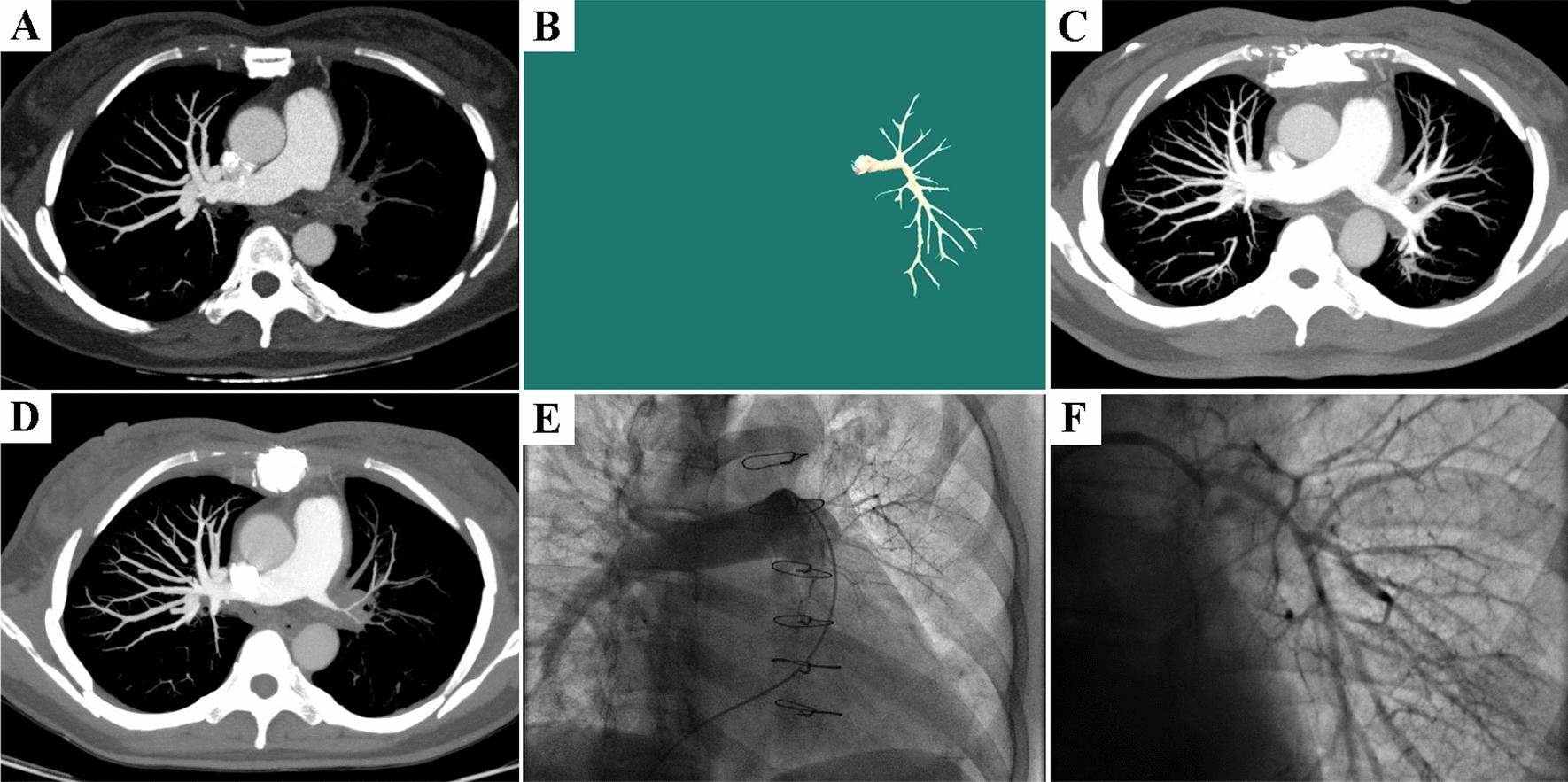
Imaging data of case 1: **A** preoperative CTPA showing the occlusion of the left main pulmonary artery; **B** intima tissue obtained from pulmonary endarterectomy; **C** postoperative CTPA showing significant perfusion recovery of the occlusive pulmonary artery; **D** CTPA at 4 months after pulmonary endarterectomy showing restenosis; **E**, **F** subsequent balloon pulmonary angioplasty for relieving restenosis of the left main pulmonary artery and branches

Clinical and hemodynamic results at 3 months after PEA were assessed and compared with baseline, as indicated in Table 2. With improved NYHA functional class (*P* = 0.023) and decreased mean pulmonary artery pressure (mPAP) (33 [20–48] mmHg before versus 21 [16–26] mmHg after; *P* < 0.018), the symptoms were significantly relieved in all patients. Although no statistically significant change was found, the general numerical distribution of pulmonary vascular resistance (234 [131–843] dyn.s.cm^−5^ versus 180 [150–372] dyn.s.cm^−5^; *P* = 0.310) shifted to the left after surgery, suggesting a trend for better hemodynamics. The blocked PA corresponding to the surgical region was shown to reopen with blood filling by postoperative CTPA (Fig. 2).

### Mid-term outcomes

The median follow-up duration was 26 months until the stated expiration date. Despite continued administration of oral corticosteroids, three patients (cases 1, 3, and 4) without symptoms relapsed at 4-, 4-, and 5-month follow-up, respectively. CTPA indicated PA restenosis (Fig. 2). Initial BPA for treated or distal involved arteries was performed on these patients 5 months following PEA, after multidisciplinary discussion and patients’ informed consent was obtained. Two patients then underwent repeated BPA for multi-segmental arterial involvement (Table 2). Immediate pulmonary angiography indicated significantly relieved stenosis and improvements in perfusion in the main, lobar, and segmental PA. These patients are now symptom-free and regularly visit the clinic. In addition, one asymptomatic patient (case 5) without visible disease progression underwent BPA once for distal stenosis.

## Discussion

The current study found two important findings in patients who underwent PEA for obstructive IPV. First, PEA alone or with patch angioplasty is safe and feasible to relieve occlusion caused by IPV, and PEA combined with subsequent BPA might be a promising therapeutic option. Second, comprehensive treatment protocols after PEA, including medications beyond the conventional dosage regimen of corticosteroids, should be investigated to improve long-term outcomes.

Studies on IPV are limited; only a few studies, particularly those that focus on the use of PEA in patients with IPV, have been conducted [2–4, 7]. However, because the PEA techniques for IPV are similar to those for CTEPH, previous results in patients with CTEPH were helpful as a point of comparison. Under skilled hands, PEA, which has been refined, assessed, and used as the gold standard treatment for individuals with CTEPH for decades, could be potentially curative and safe [8]. According to reports from the University of California-San Diego Medical Center where the PEA technique was pioneered and developed, perioperative mortality decreases from 20% in the early stages to 2%, with 5- and 10 year survival rates of 82% and 75%, respectively, which were significantly improved compared with those who did not receive PEA [8, 11]. Based on the most recent similar results from our center, which have become better over time, the perioperative death rate was 1.2% (2/171) in 2015, with a 10-year survival rate of 83.9% [10]. Similar to the prior CTEPH group, the majority of the patients in the current cohort did not have postoperative complications, including reperfusion pulmonary edema, pulmonary bleeding, delirium, or lung infection, regardless of whether an additional patch expansion of PA was performed. These advancements might be attributed to the accumulated surgical expertise gained during the learning curve, bloodless vision with deep hypothermic circulatory arrest, enhanced perfusion strategy, and integrated management in the cardiac critical care unit [10, 12].

Unfortunately, one patient died during the postoperative period while still in the hospital because of residual PH and right heart failure. As the peripheral portion of the arterial tree accounted for more than 80% of the pulmonary arterial compliance [13] and determined the normalization of pulmonary vascular resistance [14] following successful PEA, PEA limited to the major left PA was insufficient for this patient. In our experience, arteries in the IPV were more likely to be transmurally implicated and to exhibit circumferential artery wall fusion and thickening, which correlates to the widespread lymphocytic infiltration, whereas in CTEPH, inner layer alteration was predominantly observed [10]. The PEA in IPV has occasionally been technically challenging due to this anatomical difference, particularly when surgeons attempt to reach the lobar segmental PA and separate the intima. Therefore, recognition of residual PH and pulmonary vascular resistance in patients with severe PH who underwent partial PEA is important because they are adversely related to postoperative and long-term survival [14, 15]. In this aspect, early detection and treatment might ease PEA as the inflammation might extend outward and lead to irreversible vascular changes as the day progresses [3, 4].

The United States CTEPH Registry presented declines in median mPAP from 44 to 24 mm Hg and total pulmonary resistance from 9.4 to 4.4 Wood units after PEA [16]. In contrast to CTEPH, arterial circumferential constriction in pulmonary vasculitis seems to be rather common [17], which calls for additional PA augmentation. Our team selected patch angioplasty, which was performed in four of seven patients. Alternative techniques including bypass surgery and graft replacement have also been discussed. Surgical outcomes are satisfactory, with no deaths or significant complications [6]. In any case, using these intensive therapies is preferable to leaving the occlusive PA untreated or performing lung transplantation prematurely [14]. Apart from its role in restoring the pulmonary arteries, PEA plays a pivotal role in the diagnosis of IPV. Among the patients included in the current study, no evidence of any other autoimmune disease was detected prior to the PEA procedure. It is important to note that characteristic manifestations commonly associated with vasculitis, such as multiple organ involvement, aneurysms, ulcers, fever, and specific autoantibodies, were absent, rendering them inadequate to fulfill the diagnostic criteria for vasculitis like polyarteritis and Behcet's disease. The diagnosis of IPV is usually uncertain until confirmed through biopsy, which aligns with findings reported in the majority of published studies on IPV to date [2–4].

BPA is another developing technique for restoring flow in stenotic PA in CTEPH and Takayasu arteritis [18, 19]. Two patients in this group tried interventional treatment for the first time before receiving PEA but were eventually deemed ineligible for BPA because their total PA occlusions were either not amenable to route pathway establishment or had an unfavorable risk–benefit ratio. This type of situation was first investigated by Gerges et al. [18]. The overall success rate for recanalization of 352 chronic total PA occlusions was 50% in the expert center, which was much lower than the success rate for coronary chronic total occlusions [20]. Thus, surgery with complete PEA offers several patients a chance to undergo further therapies. When PEA was successfully completed, a significant improvement in pulmonary perfusion on the BPA scan and relief of PH were observed in this study, consistent with previous reports [2, 4]. If numerous segmental involvements or recurrences develop, PEA might be a pivotal therapy in individuals with occlusive IPV before prospective BPA. Three individuals in our study agreed to undergo BPA for proximal or distal lesions. Now, their condition has stabilized.

Since IPV is typically speculated to be a progressive, relapsing disease, arterial restenosis is the most worrisome consequence of PEA. In the current study, the incidence of proximal restenosis of the treated PA within the 6 month follow-up period was high (3/6) for the first time. The amount of time before deterioration may vary significantly from months to years [2–4]. Although active inflammation may have played an important role in this result, detecting such a process at an early stage may be challenging because of the frequent mismatch between symptoms, well-known inflammatory indicators, such as ESR and CRP, and imaging [6, 21]. The strategy of monitoring IPV needs to be investigated in further research, particularly focusing on depicting preoperative subclinical inflammation that largely determines surgical timing [22].

Medications are the cornerstone of treating IPV. It is well known that anti-inflammatory therapy is crucial in the treatment of vasculitis, as it can alleviate symptoms and reduce the risk of restenosis [23]. For patients with active vasculitis, immunosuppressive therapy should be initiated first, and the timing of surgery should be determined by a multidisciplinary team after the condition has improved [24]. However, due to the nonspecific symptoms that many IPV patients present with at the onset of the disease, such as cough, difficulty breathing, and chest pain, combined with the low incidence rate, a definitive diagnosis is often delayed [4]. The preoperative diagnosis of most reported IPV cases is uncertain, with CTEPH being more frequently considered [2, 4]. A definitive diagnosis is made after biopsy, and immunosuppressive therapy is initiated at that point. Therefore, there is a need to strengthen early detection of IPV. Additionally, there is currently no well-established medication protocol for IPV patients after PEA. Patients in our cohort were prescribed oral prednisolone 60 mg daily following surgery, in accordance with the maximum initial dose recommended by the European League Against Rheumatism for active large vessel vasculitis, with a subsequent slow tapering regimen [25]. However, major relapses still occur in certain individuals at random. According to a recent study by Yanartaş et al., azathioprine seems to have an additional beneficial effect in postponing IPV restenosis [2]. Furthermore, in clinical trials, combination therapy with biologic immunosuppressants has been used for patients who are steroid-resistant or experience relapse after steroid tapering [4, 26, 27]. Despite the urgent need, there is currently no definitive evidence to suggest that a specific medication or combination therapy is superior to others in terms of inflammation suppression in the treatment of IPV.

During the chronic course, PH develops in some IPV patients as a result of progressive vascular narrowing and remodeling. In this study, one patient received a combination of tadalafil and ambrisentan during the perioperative period, resulting in a substantial reduction in both mPAP and PVR following PEA. Apart from this case, no other patients received drugs approved for pulmonary arterial hypertension. In sporadic IPV cases, bosentan, sildenafil, and epoprostenol were also used and showed positive effects [28, 29]. However, relying solely on PH-specific medications for the treatment of PH secondary to IPV may be impractical. In a recent multicenter cohort study, PH-specific medications improved the hemodynamic status of patients with Takayasu's arteritis-associated PH but did not achieve normalization [30]. In a randomized trial conducted by Bhasin et al., early administration of sildenafil increased the extent of postoperative reduction in PA pressure in patients undergoing corrective surgery for ventricular septal defect, indicating that combining multiple treatment strategies may bring greater benefits [31].

## Limitations

This study had several limitations. First, this was a small cohort study. The absence of a comparison with the non-surgery group and the limited sample sizes weakens the results to a certain degree. However, to our knowledge, our study represents the major series of occlusive IPV treated with PEA at a single institution with mid-term follow-up and is the first to reveal the incidence of relapse after surgery, given the rarity of IPV. It provides valuable therapeutic opinion for occlusive IPV and highlights the necessity of optimizing management strategies. Second, data were retrospectively collected and analyzed, thereby introducing inherent bias, although all PEA and perioperative treatments were carried out by the same team. Furthermore, the follow-up time was not long enough to assess late vascular patency and other cardiovascular events.

## Conclusions

In conclusion, the data from this series tentatively demonstrate that PEA is a feasible procedure for the treatment of occlusive IPV, with significant hemodynamic and symptomatic benefits. The combination of PEA and BPA may be a promising therapy for aggressive IPV with recurrence. However, management of long-term vascular patency still requires further improvement. Advanced monitoring strategies and immunosuppressants are potentially useful.

## Data Availability

The datasets used and/or analyzed during the current study are available from the corresponding author on reasonable request.

## Abbreviations

BPA: Balloon pulmonary angioplasty
CTEPH: Chronic thromboembolic pulmonary hypertension
CTPA: Computed tomography pulmonary angiography
CRP: C-reactive protein
ESR: Erythrocyte sedimentation rate
IPV: Isolated pulmonary vasculitis
mPAP: Mean pulmonary artery pressure
NYHA: New York Heart Association
PA: Pulmonary artery
PEA: Pulmonary endarterectomy
PH: Pulmonary hypertension
